# Burden or Blessing? CEO Early-Life Adversity Experience and Firm Internationalization Performance

**DOI:** 10.3389/fpsyg.2022.855316

**Published:** 2022-05-03

**Authors:** Ping Zhou, Yujie Zhao, Kangsheng Zhao

**Affiliations:** ^1^Innovation and Entrepreneurship Education Research Center, Guangdong University of Foreign Studies, Guangzhou, China; ^2^School of Management, Shanghai University, Shanghai, China; ^3^Department of Economic Management, Guangdong Construction Polytechnic, Guangzhou, China

**Keywords:** send-down movement, organizational discretion, institutional discretion, chief executive officers, early-life adversity, internationalization performance

## Abstract

Firm internationalization is a great challenge that needs a strong leader because of the uncertainty involved. Previous research has examined the antecedents of firm internationalization from the perspective of upper echelons theory, including chief executive officer (CEO) or top management team (TMT) characteristics, yet little research has examined the role of CEO early-life adversity experiences. Based on the imprinting theory and upper echelons theory, this study explores the relationship between CEO early-life adversity experience and firm internationalization. Using data from Chinese listed companies during the period 2007–2017, we demonstrate that CEO early-life adversity experiences have a significantly positive effect on firm internationalization; the effect is stronger when the CEO with early-life adversity has a higher level of organization discretion and institutional discretion. The findings are robust to a battery of alternative variable definitions and estimation methods. We contribute to upper echelons theory and the research on internationalization antecedents by showing that CEO early-life adversity has a positive effect on firm internationalization. The findings suggest boards aspiring to expand internationally place weight on candidates’ early-life experiences while selecting and hiring CEOs and confer them managerial discretion to better play their strength after CEO’s appointment decision is made.

## Introduction

The past 30 years have seen a substantial increase in industrial exploration and scholarly interest in internationalization because of advancements in communication and transportation technologies. Internationalization helps firms obtain a variety of benefits, such as gaining technological and managerial knowledge spillover, inspiring innovative product ideas, expanding business portfolio and developing future revenue streams, and increasing competitive advantage, However, internationalization also means huge risks compared to general market expansion. In the field of international business research, the driving factors of firms’ internationalization strategy-making and performance have long been hot issues.

Because of the great uncertainty along with internationalization process, researchers tend to believe that firm internationalization requires strong leaders, and some scholars have analyzed the impact of chief executive officer (CEO) or TMT traits on firm internationalization and performance from the perspective of upper echelons theory. For instance, Jaw and Lin (2009) claim an inverted U curvilinear relationship between CEO position tenure and the firm’s internationalization. Camino Ramon-Llorens et al. (2017) show that the level of success in internationalization is influenced by CEOs’ academic level of achievement using survey data from Spanish family firms. Not only has the influence of CEO explicit traits on firm internationalization been tested, but also the impact of psychological traits such as CEO overconfidence (Zhong et al., 2018), CEO openness (Chen Z. H. et al., 2020), etc. has been explored. Yet those studies investigating the role of CEO personality on firm internationalization have not analyzed how CEOs’ psychological traits are shaped by their experiences. So we know little about how CEOs’ early-life experiences influence firm internationalization performance. For example, it remains unknown who is better suited to take a company aspiring to internationalize to success: an over-confident leader with relatively smooth experience or a leader who has experienced adversities in his or her early life? Moreover, prior studies have mostly used proxy variables to measure CEO psychological traits because it is difficult to ask CEOs to fill out questionnaires. And the archive data from which those proxies were derived from in early-stage research was quite limited, so the measurement of psychological traits was indirect and imperfect although the researchers did their best to search for reasonable proxies (Lian et al., 2015). With more information disclosed to the public, we are now able to examine the impact of CEO early-life experience on firm internationalization performance with data of higher quality.

On the other hand, the stream of literature concerning CEO early-life experience mainly explored CEO experience shaped by natural, economic events, yet little research investigates the impact of political and social events. For example, Bernile et al. (2017) show that CEOs who experience natural disasters without hugely negative results behave more aggressively in firm strategy-making, whereas CEO early-life exposure to natural disasters with extreme disadvantages makes them more conservative in the behavior. Malmendier and Nagel (2009) find that CEOs who lived through the Great Recession tend to maintain robust capital structure with less leverage, and those who started their careers during the recession have similar risk preferences. O’Sullivan and Zolotoy (2021) argue and test the positive effect of CEO early-life disaster experience on corporate social performance through the theoretical lens of post-traumatic growth. Xu and Li (2016) find that firms led by CEOs born in poverty counties during “the Great Famine” made more charitable donations. Although these studies help elucidate the impact of top managers’ early life experience on their beliefs and values and firm outcomes, we know little about the role of CEO early-life adversity instigated by social disorder on firm strategy. As more and more experience in dealing with natural disasters and economic turbulences has been accumulated, people are more capable of dealing with those incidents, thus the negative impact of such incidents becomes more predictable. In contrast, there is much less knowledge on dealing with political trauma on the individual level because fewer political events affecting the general public have been accurately recorded and narrated. So it is worthwhile to explore the impact of the social disorder on individuals and society. After all political and social disorders still occur across the world and influence the youths profoundly. How early life adversity instigated by social events influences the experiencers’ characteristics and subsequent outcomes is worthy of exploration.

To fill these gaps, we examine the relationship between CEO early life adversity and firm internationalization performance based on imprinting theory and upper echelons theory. We also investigate the moderating role of internal organizational and external institutional conditions on the main effect. The study shows that the listed companies led by CEOs with sent-down experience have better internationalization performance. And the positive impact of CEO sent-down experience on firm internationalization is strengthened by both organizational discretion and institutional discretion. Our findings are robust after controlling for firm fixed effects, different measures of early life adversity, propensity score matching, and two stage Heckman model.

Our study makes several notable contributions. First, the study enriches upper echelons theory by extending the research on CEOs’ early-life experiences. Prior studies mainly explore the impact of CEO traits shaped by their experience of natural disasters and economic depression on firm strategies and organization outcomes, little is concerned about the early-life adversity instigated by social disorder. Our study links Chinese CEO sent-down experience to their self-efficacy in dealing with uncertainty and change and further to firm internationalization performance, thereby complementing research on CEOs’ early-life experiences. Second, our research adds to research on the antecedents of firm internalization from the perspective of the upper echelons theory by revealing the positive impact of CEO early-life adversity experience on firm internationalization. Third, our study also contributes to the existing studies on the historical event of the send-down movement by extending the consequences of individual sent-down experience to the firm level.

The rest of the paper is organized as follows: the second part mainly reviews the relevant literature and proposes the research hypotheses; the third part is the data source and research design, which introduces the measurement and model design; the fourth part is the empirical results and analysis; the fifth part is the research conclusion and managerial implications.

## Literature Review and Hypothesis Development

### Sent-Down Experience and Its Imprint on Individuals

In 1955, Chairman Mao Zedong proposed that “the countryside is a vast world where much can be done,” which became the slogan of the send-down campaign (also known as the “up to the mountains and down to the countryside” movement). On 22nd December 1968, Chairman Mao Zedong instructed People’s Daily to write an article entitled “We also have two hands, do not idle in the city,” and encouraged the educated youth to move to the countryside and be educated by the rural workers and farmers.

After that, secondary school graduates (who were regarded as educated youth then) were systematically assigned to the countryside. The send-down campaign lasted 25 years and involved a total of about 20 million young people. The movement was mandatory and involved nearly all urban families, thus resulting in a large number of sent-down youths (referred to as “Zhiqing” in Chinese) (Roland and Yang, 2019). The city-bred educated youths across China aged from 14 to early 20s were deprived of opportunities of formal education and sent to the countryside and assigned to live with the peasants to farm for a living. Sent-down youths could not choose whether to go to the countryside or where they would stay. Except for special circumstances, sent-down youths had to toil more than 2 years before they were allowed to join the army, factories, or pursue further education.

Elder and Glen (1986) point out that youth is the most crucial period to form preferences for individuals, and that major events in youth, such as economic and political system changes and macro-economic turbulence, have a lasting impact on the psychological characteristics of the experiencer. As one of the largest and most far-reaching events in modern Chinese history, the send-down campaign greatly influenced the generation born from 1946 to 1961. They were forced to go to remote rural regions and be “re-educated” by farmers from the bottom of society, undergoing harsh manual work and having no idea whether or when they could go back. At an early stage of life, sent-down youths had to leave their families and experience severe poverty, extreme backwardness, and brutal competition for quotas to go back to the city. They had to develop special skills to cope with the challenging living conditions and uncertain future (Shi and Zhang, 2019).

Prior studies have displayed the impact of the Send-down Campaign on individual level and social level not only in a negative tone but also in a positive tone. For example, Liang and Li (2014) investigate the impact of sent-down experience on social trust and find that the degree of distrust of experts and scholars among people with sent-down experience is significantly higher than others. Shi and Zhang (2019) state that the sent-down experience lessens individuals’ participation in politics. However, some studies point out that the sent-down experience also led to some positive side effects. Jia et al. (2018) find that sent-down experience had a positive impact on the experiencer’s college admission test results after the resumption of the college entrance exam because it allowed sent-down youths to develop special cognitive abilities to cope with harsh environments. The youth who were sent away from their families had to develop the skills to adapt to the adversity and uncertain future to survive the forced arrangement out of their control. Chen and Cheng (1999) point out that, for sent-down youths, a spirit of self-striving was developed, which not only helped them overcome the negative impact of the send-down life but also supported them in their career development or social mobility. Their viewpoints are echoed by Chinese general secretary Xi Jinping, who noted in *Xi Jinping’s 7-year sent-down life* that *“When I came to The Yellow Land at the age of 15, I was confused, and when I left the Yellow Land at 22, I had a firm goal in life and full of confidence.”*

Although the impact of sent-down experience on individual and social level has been explored, how the traits imprinted by the particular early-life adversity affect strategic decision-making in the firms led by CEOs with sent-down experience is still unknown. In this study, we examine whether CEO sent-down experiences affect firms’ internationalization from the perspective of imprinting theory and upper echelons theory.

### Chief Executive Officer Sent-Down Experience and Internationalization Strategy

Although internationalization helps firms obtain various benefits such as differentiated technological and managerial knowledge and boarder market, the process of internationalization comes with risks. For instance, internationalization not only increases the difficulties of organizational management but also means resource commitment and the challenges of high uncertainty (Hitt et al., 2009). In the field of international business, a lot of research has explored the antecedents of firm internationalization from upper-echelons perspectives. Yet the existing research has mainly explored the impact of top leaders on internationalization strategy from explicit characteristics such as CEO gender (Ng and Sears, 2017) and CEO tenure (Maitland and Sammartino, 2015), limited research has investigated the role of top managers’ psychological traits or early-life experience on the focal firms’ internationalization.

The imprinting theory, which has its roots in biology, suggests that there are “environmentally sensitive periods” in the development of an individual or an organization, in which stage the individual or organization would be impacted significantly by the environment (Pieper et al., 2015; Khan et al., 2020). Marquis and Tilcsik (2013) further claim that the imprinting of a focal actor is the result of multiple sensitive periods and that new imprinting is constantly added on previous imprinting, resulting in a dynamic change. The imprinting theory has become an important perspective that has been widely used in studies on organizational theory, entrepreneurship research, individual career development, etc. (Mathias et al., 2015; Wyrwich et al., 2022). Scholars have conducted studies from different units of analysis, such as individuals, teams, organizations, and industry clusters applying imprinting theory, which represents a theoretical lens to understand the factors influencing individual decisions (Hu et al., 2022).

Imprinting theory proposes that the traits which are formed in early life may last into adulthood. To be specific, there are three elements of imprinting: (1) the focal subject has a sensitive period, typically the founding stage of an organization or the early stage of the individual’s career; (2) the focal subject in the sensitive period will develop the corresponding characteristics to adapt to the environment; (3) these characteristics and abilities will continually affect the focal actor’s decision-making process even if environmental constraints disappear (Marquis and Tilcsik, 2013). China underwent a drastic social transformation after the founding of the country, and a significant number of today’s entrepreneurs and corporate executives experienced the send-down movement at the beginning of their careers. Therefore, the social movement that occurred during their early years would have lasting imprints on their characteristics and values. Then the imprints will manifest in firms they lead according to upper echelons theory.

On the basis of prior studies, we propose that there are two major imprints of the sent-down experiences of senior executives in their sensitive period which are highly relevant to firms’ internationalization. One is the tolerance of uncertainty. In their youth, sent-down youths had barely any control over their future in that political campaign, as it was mandatory for all urban families. They had no idea where they were going to be sent, whether and when they were able to go back to the city (Liu, 2009). The experience forced them to develop the ability to deal with life and work under highly uncertain circumstances, which helped train their tolerance of uncertainty. It is this trait that is the basis of their openness to firm internationalization strategy, as there is always a high level of uncertainty involved in firm internationalization strategy (Tihanyi et al., 2000).

The second major imprint is their self-efficacy in coping with change. At the crucial stage of their growth, the sent-down youths underwent the huge transition from town to countryside, from mental activity to tough manual labor. The tough sent-down experience developed their self-efficacy in dealing with change, leaving a deep imprint on their characteristics and personal values. As noted by Chen and Cheng (1999), “the sent-down youth may be more adaptive, as their unusual experiences probably improved their social skills and increased their political flexibility.” Those sent-down youth who just graduated from middle school had to adapt to the circumstances in those remote areas and integrate into the local community, which was completely different from their previous lives. This sudden change during their youth forced them to develop independent personalities and improve their adaptability to survive and strive for the limited opportunities to return to cities. According to imprinting theory, the characteristics developed in a sensitive period would influence the experiencers’ beliefs and coping styles permanently (Dai et al., 2018). Therefore, CEOs with sent-down experience may exhibit a high tolerance for uncertainty and the self-efficacy to deal with change in their managerial jobs.

Upper echelons theory states that an organization’s actions may reflect the personalities, experiences, and values of its top leaders (Shah et al., 2019). A leader’s coping style and personal values act as filters to analyze complex situations, which therefore influence their strategic choices and corporate outcomes (Finkelstein et al., 2009; Sarfraz et al., 2019; Khan et al., 2021). In the search for strategic opportunities, executive capabilities and coping patterns shaped by their experience matter. Combining the perspective of imprinting theory and upper echelons theory, we infer that CEOs’ tolerance for uncertainty and self-efficacy in coping with change imprinted by their sent-down experience may play an important role in predicting firms led by sent-down CEOs to seek international entrepreneurial opportunities. Therefore, we present the following hypothesis:

H1: *CEO sent-down experience has a positive impact on firm internationalization.*

Upper echelons theory holds that the effects of top managers on strategic decision-making are not only dependent on their personalities and values but also subject to the decision-makers’ latitude of autonomous action, i.e., the managerial discretion (Li and Tang, 2010). This suggests that it is necessary to incorporate the moderating role of managerial discretion when exploring the relationship between CEO sent-down experience and the internationalization performance of firms led by them. Managerial discretion determines the degree of influence that top managers have on organizational strategies and results, and is subject to various factors within and outside organizations. These are referred to as organizational discretion and institutional discretion respectively in this paper.

### The Moderating Role of Organizational Discretion

The characteristics of the organization itself either inhibit or enhance managerial discretion (Quigley and Hambrick, 2012). Two of the most important factors within an organization that affects managerial discretion are organization inertia and resource slack. The inertial forces within an organization determine the operation mode and the evolutionary path of the enterprise to some extent. The higher level the organization inertia is, the more restraints to initiate strategic changes top managers have. Organization inertia is often manifested in firm age and firm size, as prior studies point out that the larger the organization is and the longer it lives, the more practice it accumulates and the severer the degree of organizational inertia (Lian et al., 2015).

To implement internationalization strategies, the firm needs to commit resources to develop new markets and cope with uncertain situations, so resource slack determines the level of fault tolerance of strategic exploration. Therefore, resource slack is another important factor affecting the managers’ organizational discretion (Wangrow et al., 2015). The more sufficient resources within the organization, the more latitude top managers have, and the higher the possibility for them to engage in international exploration.

If managers are less constrained by organizational inertia or have more disposable organizational resources, the imprint of their early experience will be more reflected in firm internationalization strategy. Thus, we present the following hypotheses:

H2: *The positive relationship between CEO sent-down experience and firm internationalization is strengthened when organizational discretion is higher.*
H2a: *The positive relationship between CEO sent-down experience and firm internationalization is strengthened when the level of organizational inertia is lower.*
H2b: *The positive relationship between CEO sent-down experience and firm internationalization is strengthened when there is more organizational slack.*

### The Moderating Role of Institutional Discretion

The institutional environment in which the managers are embedded also affects the managers’ latitude of action (Finkelstein and Hambrick, 1990). The institutional environment of state-owned enterprises (SOEs) and regional institutional ecosystems are the two important factors that affect the institutional discretion of managers (Lian et al., 2015), which determine the latitude of managers’ decision-making externally and affect the ability and freedom of managers to choose internationalization strategies.

For SOEs, their role is not only to achieve economic growth but also to take on some political tasks and social functions, such as providing employment and stability (Chen et al., 2016). These factors cause the government to impose formal and informal institutional constraints on managers of SOEs, which ultimately restrain managerial discretion. In the process of balancing the interests of multiple stakeholders, SOEs are more likely to require managers to ensure the stability of the organization compared to non-SOEs. Therefore, managers of SOEs who intend to implement risky strategies would face more restraints.

The regional institutional environment is also an important element that restrains managerial discretion. As noted by Dai et al. (2019), the maturity of formal systems between regions is extremely uneven. Social trust, government intervention, the level of financial development, judicial justice, and other factors influencing the business environment are varied in different regions of China (Dai et al., 2018), which result in different levels of resource availability. These differences lead to the variation of CEOs’ managerial discretion, depending on where their firms are located. In regions with a better institutional environment, the advantages of high-level government efficiency, policy transparency and better financing availability equip managers with a higher level of managerial discretion. In these circumstances, CEO characteristics manifest themselves more significantly in firm strategies and organization outcomes, including internationalization. Thus, we posit:

H3: *The positive relationship between CEO sent-down experience and firm internationalization is strengthened when institutional discretion is higher.*
H3a: *The positive relationship between CEO sent-down experience and firm internationalization is more significant in non-SOEs.*
H3b: *The positive relationship between CEO sent-down experience and firm internationalization is more significant when the institutional environment the focal firm embedded in is better.*

**FIGURE 1:**
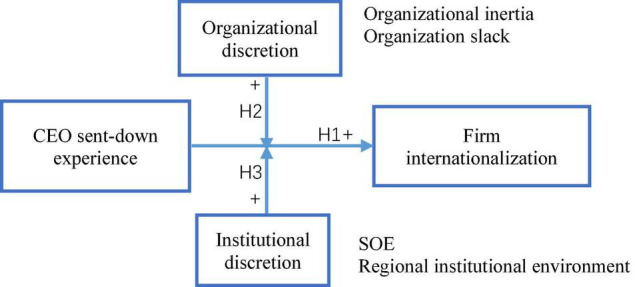
Theoretical framework.

Figure 1 shows a theoretical model summarizing the hypotheses.

## Materials and Methods

### Sample and Data

This paper explores the impact of CEO sent-down experience on firms’ internationalization. We used the China Stock Market and Accounting Research (CSMAR) database, which has been widely used in research on China’s listed firms (Wang and Qian, 2011). Our sample started with all Chinese firms listed on the Shenzhen or Shanghai stock market during 2007–2017. The following processing procedures were performed on the initial data: (1) remove listed companies in the financial and insurance industries following prior research, because these industries are highly regulated in China; (2) remove samples with missing financial and corporate governance data; and (3) winsorize all continuous variables at the 1 and 99% levels to eliminate the effect of extreme outliers. At last, we collect a total of 5,862 firm-year observations during this period. The table of industry-year distribution of the sample can be seen in Appendix A.

### Measures

#### Dependent Variable

##### Internationalization Scope and Speed

Following Zhong et al. (2018), we measured firms’ internationalization performance by the scope and speed of their internationalization process. *Scope* is the number of countries that a firm established subsidiaries in during its internationalization. *Speed* is denoted by the 5-year average growth ratio of the international revenue for the focal firm. In line with the existing research (Chen et al., 2016), we first regressed the natural logarithm of a focal firm’s international revenue and an index variable of the time in years, with time serving as an independent variable. Then, the antilog of the regression coefficient (b2), capturing the growth rate of the firm’s international revenue, was used as the measurement of its pace of international expansion. The basic growth equation for this measure is given by Ln(INT_REVENUE_t_) = b1+b2t+δ. INT_REVENUE_t_ is a firm’s international revenue in year *t*; δ is residual; the antilog of b2 is the speed of internationalization.

#### Independent Variable

##### Sent-Down Experience

If the CEO of a sample firm has sent-down experience, then *sentdown* takes the value of 1, and it takes 0 otherwise. We collected the CEO names and birth years of all sample firms, then we manually searched for detailed resume information from Baidu Encyclopedia, Hexun News, and the Encyclopedia of Financial Affairs and Encyclopedia. If a CEO’s resume from 1968 to 1976 includes experiences such as “Zhiqing” (educated youth), “farm,” “production and construction corps,” “commune,” we inferred his or her experience as a possible sent-down youth. We then matched the resume information with the relevant information of China Youth Network to verify whether the CEO had been in the send-down movement. If the CEO had experienced the send-down movement, we confirmed that the CEO has sent-down experience.

#### Moderating Variables

Our hypotheses 2 and 3 predict that the positive relationship between CEO’s sent-down experience and firm internationalization is strengthened for the CEOs with a higher level of organization discretion and institutional discretion respectively.

##### Organization Discretion

We used *organization inertia* and *organization slack* to measure organization discretion. Organizational inertia greatly limits managers’ freedom of choice within the organization. The greater the organizational inertia, the lower level of organization discretion the top managers have, so organizational inertia is a reverse index of organization discretion. Based on previous research (Finkelstein and Hambrick, 1996; Li and Tang, 2010), we use *firm size* and *firm age* to measure the degree of organizational inertia. The bigger size and the older firm indicate a higher level of organizational inertia, thus less organization discretion for senior executives.

Organizational slack. The more abundant resources are available to top managers, the greater the top managers’ autonomy in strategic exploration. The disposable resources reflect top managers’ organization discretion from a different perspective. Drawing on previous research (Lian et al., 2015; Tang et al., 2015), we measure *slack* by equity/debt ratio.

##### Institutional Discretion

We use *SOE* and *Market* to measure CEOs’ institutional discretion.

(1) SOE constraints (*SOE*). Due to government intervention and their diverse objectives, the SOEs constrain the freedom of managers. Compared with non-SOE managers, the latitude of managers in SOE is relatively small, so *SOE* could be used to denote the institutional discretion imposed on top managers. *SOE* is a dummy variable that takes the value of 1 if the company is state-owned, and 0 otherwise.
(2) Regional marketization degree (*Market*). This study uses the Chinese marketization index of Wang et al. (2017) to measure institutional discretion. The index is widely used to denote the regional level of marketization (Dai et al., 2016). The worse the institutional environment that the focal firm is embedded in, the less discretion the managers have. If a firm is located in an area with a marketization index reading equal to or above the median value, the firm is placed into the high marketization group. In this case, the variable *market* equals 1; otherwise, it is put in the low marketization group, and *market* equals 0.

#### Controlled Variables

To rule out other explanations, our study uses individual-, firm-, and industrial-level variables as controls.

At the individual level, we controlled for CEO gender, level of education, and tenure. As Hitt et al. (2009) state, the traits of top managers influence firm internationalization. Thus, we controlled for CEO demographic traits.

At the firm level, we controlled for firm characteristic and governance characteristics variables including firm size, leverage, Top 1 shareholder, duality, board size, and independent director ratio. The larger the firm, the broader resources that can be used, and there is a potential positive impact of firm size on internationalization. Leverage reflects the possible constraints of creditors on firm internationalization. Top 1 is the ratio of the number of shares that the largest shareholder holds, representing the effect of large shareholders on firm internationalization. Duality, board size, and independent director ratio denote the level of corporate governance and reflect the effects of corporate governance factors on firm internationalization.

At the industrial level, we control for industry growth and industry competitiveness. Industry growth may influence firms’ international expansion prospects. We use industry concentration of sales to reflect the degree of competition in the industry which may affect a company’s willingness and ability to engage in internationalization. All variables (as well as the controls) are defined in the Appendix B.

### Estimation Methods

To test the hypotheses, we use the following regression models:


(1) $$\begin{array}{c} Scope_{t}/Speed_{t}=\alpha +\beta_{1}Sentdown_{t-1}+\beta_{2}X_{t-1}+Year\\ \;+Industry+\varepsilon_{t} \end{array}$$

(2) $$\begin{array}{c} Scope_{t}/Speed_{t}=\alpha +\beta_{1}Sentdown_{t-1}+\beta_{2}Sentdown_{t-1}^{*}\\ \;Size_{t-1}/Fage_{t-1}+\beta_{3}Size_{t-1}/Fage_{t-}\\ \;+\beta_{4}X_{t-1}+Year+Industry+\varepsilon_{t} \end{array}$$

(3) $$\begin{array}{c} Scope_{t}/Speed_{t}=\alpha +\beta_{1}Sentdown_{t-1}+\beta_{2}Sentdown_{t-1}^{*}\\ \;Slack_{t-1}+\beta_{3}Slack_{t-1}+\beta_{4}X_{t-1}+Year\\ \;+Industry+\varepsilon_{t} \end{array}$$

(4) $$\begin{array}{c} Scope_{t}/Speed_{t}=\alpha +\beta_{1}Sentdown_{t-1}+\beta_{2}Sentdown_{t-1}^{*}\\ \;SOE_{t-1}+\beta_{3}SOE_{t-1}+\beta_{4}X_{t-1}+Year\\ \;+Industry+\varepsilon_{t} \end{array}$$

(5) $$\begin{array}{c} Scope_{t}/Speed_{t}=\alpha +\beta_{1}Sentdown_{t-1}+\beta_{2}Sentdown_{t-1}^{*}\\ \;Market_{t-1}+\beta_{3}Market_{t-1+}\beta_{4}X_{t-1+}Year\\ \;+Industry+\varepsilon_{t} \end{array}$$

*Scope_*t*_/Speed_*t*_* are dependent variables, that designate firm internationalization performance. *Sentdown* represents CEO sent-down experience. Moderators include the contingent factors mentioned in the hypotheses, including organization inertia, organization slack, SOE, and regional institutional environment. *X*_*t*–1_ represents the control variables at time period *t-1* as mentioned above. We also include industry fixed effect and year fixed effect. Eq. (1) tests the effect of CEO sent-down experience on firm internationalization performance. Eq. (2) and Eq. (3) test the moderating effect of organization discretion from the perspective of inertia and slack respectively. Eq. (4) and Eq. (5) focus on the moderating effect of institutional discretion from the perspective of SOE and regional institutional environment respectively. The *t*-statistics (in parentheses) are based on standard errors clustered by firm.

## Results

### Descriptive Statistics

Table 1 presents the descriptive statistics. The mean value of sent-down experience is 6.76%, which indicates that 6.76% of CEOs in the sample have sent-down experience. 30.93% of the sample companies are SOEs.

**TABLE 1:** Descriptive statistics.

Variables	Mean	Median	STD	P1	P25	P75	P99
*Scope*	2.5242	2.0000	3.0987	1.0000	1.0000	3.0000	14.0000
*Speed*	1.1578	1.1554	0.2849	0.5016	0.9697	1.3547	1.8133
*Sentdown*	0.0676	0.0000	0.2510	0.0000	0.0000	0.0000	1.0000
*Sentdown2*	0.0542	0.0000	0.2265	0.0000	0.0000	0.0000	1.0000
*SOE*	0.3093	0.0000	0.4622	0.0000	0.0000	1.0000	1.0000
*Fage*	1.8784	1.9459	0.8745	0.0000	1.3863	2.6391	3.1781
*Slack*	2.5522	1.3861	4.4958	0.1834	0.7334	2.7411	17.7617
*Market*	0.6571	1.0000	0.4747	0.0000	0.0000	1.0000	1.0000
*Size*	22.2515	22.0611	1.3147	19.9627	21.3128	22.9506	26.2689
*Lev*	0.4243	0.4191	0.1984	0.0533	0.2673	0.5769	0.8450
*Top1*	0.3532	0.3436	0.1426	0.0912	0.2427	0.4579	0.7556
*Board*	2.2554	2.3026	0.1749	1.7918	2.0794	2.3026	2.7726
*Indep*	0.3759	0.3529	0.0554	0.3333	0.3333	0.4286	0.5714
*Dual*	0.3050	0.0000	0.4605	0.0000	0.0000	1.0000	1.0000
*Tenure*	3.4795	3.5835	1.0180	0.0000	2.9444	4.2767	5.0689
*Edu*	2.3651	3.0000	1.7771	0.0000	0.0000	4.0000	5.0000
*Gender*	0.7605	1.0000	0.4268	0.0000	1.0000	1.0000	1.0000
*Indusgrow*	0.0387	0.0465	0.2896	−0.6299	−0.0613	0.0465	1.2658
*IndusH*	0.8675	0.9174	0.1326	0.1887	0.8420	0.9496	0.9792

We present the correlation coefficients in Table 2. Consistent with H1, sent-down experience is positively correlated with international scope and speed respectively (*r* = 0.297, *p* < 0.01; *r* = 0.118, *p* < 0.01), suggesting that CEO sent-down experience is related to a higher level of internationalization. In addition, Table 2 shows that the correlations among the key variables are not very high, indicating that multi-collinearity should not be a major concern.

**TABLE 2:** Correlation matrix.

	*Scope*	*Speed*	*Sentdown*	*Sendtdown2*	*Fage*	*Slack*	*SOE*	*Market*	*Size*	*Lev*	*Top1*	*Board*	*Indep*	*Dual*	*Tenure*	*Edu*	*Gender*	*ER*
*Speed*	0.008																	
*Sentdown*	**0.297**	**0.118**																
*Sentdown2*	**0.276**	**0.125**	**0.744**															
*Fage*	**0.058**	–0.002	**0.076**	**0.048**														
*Slack*	–**0.061**	–0.019	–**0.051**	–**0.049**	–**0.266**													
*SOE*	–0.005	0.013	**0.091**	**0.094**	**0.421**	–**0.176**												
*Market*	***0.025***	–0.002	**0.018**	**0.041**	–**0.168**	**0.083**	–**0.121**											
*Size*	**0.162**	0.01	**0.136**	**0.119**	**0.442**	–**0.301**	**0.420**	–**0.131**										
*Lev*	**0.083**	0.018	**0.078**	**0.076**	**0.427**	–**0.563**	**0.361**	–**0.134**	**0.575**									
*Top1*	0.015	0.015	**0.087**	**0.080**	**0.416**	–**0.172**	**0.886**	–**0.109**	**0.433**	**0.367**								
*Board*	0.009	*0.027*	0.021	–0.009	**0.155**	–**0.134**	**0.278**	–**0.092**	**0.271**	**0.198**	**0.285**							
*Indep*	0.004	–0.012	0.025	**0.044**	–**0.055**	**0.052**	–0.007	0.019	**0.066**	–0.033	–0.02	–**0.486**						
*Dual*	0.012	0.003	–***0.022***	0.003	–**0.230**	**0.135**	–**0.301**	**0.111**	–**0.208**	–**0.186**	–**0.281**	–**0.204**	**0.090**					
*Tenure*	**0.044**	0.016	–0.001	0.015	**0.123**	–0.001	–**0.120**	0.007	*0.031*	–*0.031*	–**0.118**	–**0.057**	0.016	0.139				
*Edu*	0.004	0.009	0.014	***0.022***	–**0.119**	**0.051**	**0.034**	**0.075**	–0.014	–0.025	**0.050**	**0.055**	0.023	0.006	–0.018			
*Gender*	–**0.042**	0.002	*0.034*	**0.043**	**0.036**	*0.028*	**0.124**	**0.093**	–0.015	0.036	**0.118**	**0.098**	–0.021	–0.037	–0.094	0.581		
*Indusgrow*	–0.014	–0.012	–0.014	–0.009	–*0.029*	–0.009	–***0.022***	0.01	–0.027	0.005	–*0.030*	–0.017	0.004	0.02	–0.044	–0.016	–0.056	
*IndusH*	–**0.049**	0.014	–0.016	–***0.022***	**0.071**	–*0.030*	–***0.024***	–*0.029*	–0.066	–**0.042**	–0.006	0.005	–0.007	0.034	0.075	–0.003	0.01	–0.005

*This table presents the Pearson’s correlation, for the dependent and independent variables. Bold, bold-italicized, and italicized correlations represent statistical significance at 1, 5, and 10%, respectively. All variables are defined in Appendix B.*

### Test of Hypotheses

#### Main Effect

Hypothesis 1 predicts that the CEO sent-down experience is positively correlated with firm internationalization. In Model (1) (2) of Table 3 the dependent variable is internationalization scope, and Model (2) includes all the control variables. The results show that sent-down experience is positively related to *Scope* (β = 3.537, *t* = 5.04). In Model (3) and (4), the dependent variable is internationalization speed, and according to the results, the positive coefficient of sent-down experience is still significant (β = 0.144, *t* = 7.81), indicating that CEO sent-down experience strengthens the degree of firm internationalization. H1 is supported.

**TABLE 3:** Sent-down experience and firm internationalization.

	(1) Scope	(2) Scope	(3) Speed	(4) Speed
*Sentdown*	3.764***	3.537***	0.142***	0.144***
	(5.63)	(5.04)	(7.63)	(7.81)
*Size*		0.342***		−0.007*
		(3.87)		(−1.67)
*Lev*		0.317		0.033
		(0.90)		(1.39)
*Top1*		–0.286		0.007
		(–1.54)		(0.66)
*Board*		–0.187		0.058**
		(–0.43)		(2.24)
*Indep*		–1.443		0.030
		(–1.30)		(0.40)
*Dual*		0.141		0.006
		(1.04)		(0.72)
*Tenure*		0.023		0.001
		(0.45)		(0.31)
*Edu*		0.092**		–0.000
		(1.98)		(–0.09)
*Gender*		–0.214		–0.034*
		(–0.72)		(–1.69)
*Indusgrow*		–0.149		–0.007
		(–1.05)		(–0.57)
*IndusH*		–0.945		0.026
		(–1.60)		(0.72)
*_cons*	1.549***	–4.095**	1.106***	1.103***
	(4.81)	(–2.13)	(29.73)	(10.02)
*Industry*	Yes	Yes	Yes	Yes
*Year*	Yes	Yes	Yes	Yes
*N*	5862	5862	5862	5862
*adj. R^2^*	0.105	0.123	0.105	0.116

*p < 0.1, **p < 0.05, and ***p < 0.01 denote statistical significance.

*The t-statistics (shown in parentheses) are reported based on standard errors clustered at the firm level. Variables are winsorized at the 1% level. All variables are defined in Appendix B.*

#### The Moderating Effect of Organizational Discretion

Hypothesis 2 predicts that organizational discretion will moderate the positive correlation between CEO sent-down experience and firm internationalization. We use organizational inertia and organizational slack to indicate organizational discretion, and we test their moderating effects in Tables 4, 5 respectively.

**TABLE 4:** Sent-down experience, organizational inertia and enterprise internationalization.

	(1) Scope	(2) Scope	(3) Speed	(4) Speed	(5) Scope	(6) Scope	(7) Speed	(8) Speed
*Sentdown*	37.622***	47.935***	0.834***	0.831***	0.793***	0.792***	0.228***	0.220***
	(3.00)	(3.86)	(3.07)	(2.95)	(4.10)	(3.83)	(4.74)	(4.53)
Sentdown*Fsize	–1.478***	–1.944***	–0.030**	–0.030**				
	(–2.76)	(–3.66)	(–2.55)	(–2.45)				
*Fage*					0.061***	0.017	–0.003	–0.008
					(4.86)	(1.19)	(–0.70)	(–1.44)
Sentdown*Fage					–0.149*	–0.175*	–0.042**	–0.038*
					(–1.74)	(–1.96)	(–2.07)	(–1.87)
*Size*		0.559***		–0.004		0.105***		–0.004
		(9.02)		(–0.86)		(7.99)		(–1.01)
*Lev*		0.173		0.031		0.078		0.038
		(0.49)		(1.30)		(1.14)		(1.54)
*Top1*		–0.316*		0.006		–0.096***		0.012
		(–1.66)		(0.62)		(–2.79)		(1.18)
*Board*		–0.349		0.055**		–0.069		0.050*
		(–0.81)		(2.18)		(–0.85)		(1.95)
*Indep*		–0.876		0.039		–0.160		0.016
		(–0.82)		(0.51)		(–0.69)		(0.21)
*Dual*		0.092		0.005		0.021		0.004
		(0.71)		(0.63)		(0.88)		(0.44)
*Tenure*		0.025		0.001		0.010		0.004
		(0.49)		(0.32)		(1.12)		(0.88)
*Edu*		0.085*		–0.000		0.017**		–0.001
		(1.91)		(–0.14)		(2.14)		(–0.22)
*Gender*		–0.187		–0.033*		–0.044		–0.033
		(–0.65)		(–1.66)		(–0.79)		(–1.60)
*Indusgrow*		–0.151		–0.007		–0.022		–0.009
		(–1.12)		(–0.58)		(–1.04)		(–0.70)
*IndusH*		–1.108**		0.023		–0.187*		0.021
		(–1.98)		(0.66)		(–1.84)		(0.59)
*_cons*	1.508***	–8.432***	1.105***	1.036***	0.861***	–0.925***	1.106***	1.070***
	(4.75)	(–5.13)	(29.52)	(9.41)	(9.60)	(–2.61)	(27.40)	(9.53)
*Industry*	Yes	Yes	Yes	Yes	Yes	Yes	Yes	Yes
*Year*	Yes	Yes	Yes	Yes	Yes	Yes	Yes	Yes
*N*	5862	5862	5862	5862	5862	5862	5862	5862
*adj. R^2^*	0.142	0.182	0.106	0.125	0.108	0.162	0.105	0.118

t-Statistics in parentheses, *p < 0.1, **p < 0.05, ***p < 0.01.

**TABLE 5:** Sent-down experience, organizational slack and enterprise internationalization.

	(1) Scope	(2) Scope	(3)Speed	(4) Speed
*Sentdown*	2.284***	1.918**	0.095***	0.097***
	(2.81)	(2.29)	(4.27)	(4.37)
Sentdown*Slack	0.845**	0.928**	0.027***	0.027***
	(2.11)	(2.25)	(3.87)	(3.81)
*Slack*	–0.046***	–0.016**	–0.001	–0.000
	(–4.26)	(–1.97)	(–1.51)	(–0.53)
*Size*		0.357***		–0.006
		(4.10)		(–1.58)
*Lev*		0.561		0.040
		(1.50)		(1.51)
*Top1*		–0.273		0.007
		(–1.46)		(0.71)
*Board*		–0.311		0.054**
		(–0.72)		(2.12)
*Indep*		–1.249		0.036
		(–1.14)		(0.47)
*Dual*		0.115		0.005
		(0.86)		(0.63)
*Tenure*		0.035		0.002
		(0.68)		(0.40)
*Edu*		0.099**		–0.000
		(2.12)		(–0.03)
*Gender*		–0.162		–0.032
		(–0.58)		(–1.59)
*Indusgrow*		–0.145		–0.007
		(–1.07)		(–0.56)
*IndusH*		–0.965		0.025
		(–1.63)		(0.71)
*_cons*	1.682***	–4.309**	1.110***	1.097***
	(5.08)	(–2.25)	(29.99)	(10.06)
*Industry*	Yes	Yes	Yes	Yes
*Year*	Yes	Yes	Yes	Yes
*N*	5862	5862	5862	5862
*adj. R^2^*	0.128	0.148	0.017	0.017

*t-Statistics in parentheses.*

*p < 0.1, **p < 0.05, ***p < 0.01.

Table 4 shows the regression results after adding the interaction of sent-down experience and firm size, sent-down experience and firm age. Model (2) with *Scope* as dependent variable shows the coefficient of Sent-down*Fsize is significantly negative (β = –1.944, *t* = –3.66) and Model (4) with *Speed* as dependent variable shows the coefficient of Sent-down*Fsize is also significantly negative (β = –0.030, *t* = –2.45). Likewise, Model (6) and (8) show that the coefficient of Sent-down*Fage is significantly negative (*p* < 0.1), which indicates that firm age negatively moderates the positive relationship between CEO sent-down experience and firm internationalization performance. That is to say, organizational inertia inhibits the relationship between CEO sent-down experience and internationalization. H2a is supported.

Models in Table 5 include the interaction between CEO sent-down experience and slack. Model (2) with *Scope* as the dependent variable shows the coefficient of Sent-down*Slack is significantly positive (β = 0.928, *t* = 2.25) and Model (4) with *Speed* as the dependent variable shows the coefficient of Sentdown*Slack is significantly positive (β = 0.027, *t* = 3.81). The result reveals that organizational slack enhances the relationship between CEO sent-down experience and internationalization performance. H2b is supported. To combine the results in Tables 4, 5, H2 is supported.

#### The Moderating Effect of Institution Discretion

Hypothesis 3 predicts that institution discretion will moderate the positive correlation between CEO sent-down experience and the degree of internationalization. We added the interaction of Sentdown*SOE and Sentdown*Market respectively.

Table 6 reports the moderating effects of SOE and regional institutional environment. Model (2) and (4) show that the coefficients of Sentdown*SOE are significantly negative (*p* < 0.1), which indicates that in SOEs the positive relationship between CEO sent-down experience and firm internationalization is weakened. Model (6) and (8) show that the coefficients of Sentdown*Market are significantly positive (*p* < 0.1), which means that the positive relationship between CEO sent-down experience and firm internationalization is strengthened for firms embedded in a better institutional environment, thus H3a and H3b are supported.

**TABLE 6:** Sent-down experience, organizational discretion and enterprise internationalization.

	(1) Scope	(2) Scope	(3) Speed	(4) Speed	(5) Scope	(6) Scope	(7) Speed	(8) Speed

	Model 1	Model 2	Model 3	Model 4	Model 5	Model 6	Model 7	Model 8
*Sentdown*	4.835***	4.667***	0.174***	0.173***	2.243**	1.997**	0.095***	0.098***
	(5.05)	(4.72)	(7.25)	(7.18)	(2.41)	(2.02)	(2.72)	(2.79)
*SOE*	0.118	–0.441	0.010	0.001				
	(0.84)	(–1.58)	(1.15)	(0.08)				
Sentdown*SOE	–2.362*	–2.512*	–0.073**	–0.066*				
	(–1.76)	(–1.87)	(–2.00)	(–1.82)				
*market*					–0.040	0.065	–0.007	–0.006
					(–0.36)	(0.59)	(–0.94)	(–0.69)
Sentdown*Market					2.206*	2.220*	0.068*	0.066*
					(1.94)	(1.89)	(1.77)	(1.71)
*Size*		0.369***		–0.006		0.346***		–0.007*
		(4.25)		(–1.55)		(3.89)		(–1.66)
*Lev*		0.311		0.032		0.404		0.034
		(0.88)		(1.37)		(1.17)		(1.42)
*Top1*		0.228		0.009		–0.307		0.005
		(0.77)		(0.59)		(–1.60)		(0.55)
*Board*		–0.301		0.054**		–0.223		0.055**
		(–0.72)		(2.10)		(–0.51)		(2.13)
*Indep*		–1.271		0.032		–1.583		0.024
		(–1.18)		(0.41)		(–1.44)		(0.32)
*Dual*		0.099		0.006		0.117		0.006
		(0.74)		(0.65)		(0.87)		(0.70)
*Tenure*		0.028		0.001		0.020		0.001
		(0.55)		(0.34)		(0.39)		(0.31)
*Edu*		0.092*		–0.000		0.090**		–0.000
		(1.95)		(–0.06)		(1.99)		(–0.12)
*Gender*		–0.214		–0.034*		–0.213		–0.033*
		(–0.73)		(–1.70)		(–0.72)		(–1.65)
*Indusgrow*		–0.151		–0.007		–0.185		–0.008
		(–1.07)		(–0.59)		(–1.33)		(–0.65)
*IndusH*		–0.998*		0.025		–0.916		0.028
		(–1.71)		(0.71)		(–1.56)		(0.77)
*_cons*	1.529***	–4.436**	1.101***	1.098***	1.606***	–4.073**	1.111***	1.113***
	(4.45)	(–2.32)	(28.74)	(9.99)	(4.94)	(–2.07)	(29.41)	(9.94)
*Industry*	Yes	Yes	Yes	Yes	Yes	Yes	Yes	Yes
*Year*	Yes	Yes	Yes	Yes	Yes	Yes	Yes	Yes
*N*	5862	5862	5862	5862	5862	5862	5862	5862
adj. *R*^2^	0.114	0.135	0.015	0.015	0.112	0.131	0.015	0.015

*t-Statistics in parentheses.*

*p < 0.1, **p < 0.05, ***p < 0.01.

Many of the control variables are largely in line with our predictions. Specifically, the coefficients on firm size are significant and positive, which indicates that larger firms are more likely to expand internationally.

### Robustness Checks

The following robust analysis process has been added to enhance the robustness of the results. (1) Considering there may be unobservable factors that affect internationalization on the firm level, we use firm fixed-effects panel regression model to control for firm’s unobserved characteristics. As shown in Column 1–4 Table 7, the regression results show that the effect of CEO sent-down remains significantly positive. (2) We use the chairpersons’ sent-down experience to measure early-life adversity experience. Since the chairperson has more influence on corporate decisions in some Chinese listed companies, we code *Sentdown2* as 1 if the chairperson of the board in a sample firm has sent-down experience and 0 otherwise. The results of the retest (Column 5–8 Table 7) show that chairperson’ sent-down experience has a consistent effect on firm internationalization.

**TABLE 7:** Robust tests: sent-down experience and enterprise internationalization.

	Fixed effect regression				Remeasurement of *sent-down*			
	(1) Scope	(2) Scope	(3) Speed	(4) Speed	(5) Scope	(6) Scope	(7) Speed	(8) Speed
*Sentdown*	2.466***	2.428***	0.223***	0.223***				
	(14.59)	(14.36)	(10.89)	(10.83)				
*Sentdown2*					3.855***	3.638***	0.165***	0.167***
					(5.72)	(5.33)	(8.53)	(8.88)
*Size*		0.495***		0.010		0.372***		–0.006
		(4.11)		(0.68)		(4.48)		(–1.48)
*Lev*		1.080**		–0.003		0.232		0.029
		(2.51)		(–0.06)		(0.67)		(1.22)
*Top1*		0.000		0.000		–0.297		0.006
		(.)		(.)		(–1.59)		(0.60)
*Board*		–0.171		–0.143**		–0.215		0.058**
		(–0.33)		(–2.28)		(–0.51)		(2.31)
*Indep*		–0.406		–0.128		–2.137*		0.001
		(–0.31)		(–0.81)		(–1.81)		(0.02)
*Dual*		–0.110		0.007		0.109		0.005
		(–0.76)		(0.38)		(0.82)		(0.53)
*Tenure*		–0.014		–0.001		0.017		0.001
		(–0.32)		(–0.28)		(0.34)		(0.21)
*Edu*		0.015		–0.003		0.095**		–0.000
		(0.37)		(–0.62)		(2.11)		(–0.03)
*Gender*		–0.137		–0.025		–0.205		–0.033*
		(–0.51)		(–0.78)		(–0.71)		(–1.66)
*Indusgrow*		–0.174		–0.012		–0.166		–0.008
		(–1.43)		(–0.84)		(–1.13)		(–0.63)
*IndusH*		–0.024		–0.046		–0.928		0.027
		(–0.04)		(–0.67)		(–1.59)		(0.75)
*_cons*	0.893	–9.583***	0.872***	1.095***	1.562***	–4.369**	1.106***	1.098***
	(0.87)	(–3.13)	(6.96)	(2.94)	(4.97)	(–2.31)	(29.68)	(10.11)
*Firm*	Yes	Yes	Yes	Yes				
*Industry*	Yes	Yes	Yes	Yes	Yes	Yes	Yes	Yes
*Year*	Yes	Yes	Yes	Yes	Yes	Yes	Yes	Yes
*N*	5862	5862	5862	5862	5862	5862	5862	5862
*adj. R^2^*	0.181	0.174	0.288	0.289	0.091	0.112	0.106	0.107

*t-Statistics is reported in brackets.*

*p < 0.1, **p < 0.05, ***p < 0.01.

To mitigate endogeneity concerns, we use propensity score matching method (PSM) and two-stage Heckman modeling to test the robustness of our findings.

(3) Propensity score matching method. CEOs with sent-down experience may be more likely to join firms with better internationalization performance, thus rendering the potential sample self-selection problem. Following Marquis and Qiao (2018), we conduct a PSM exercise to examine the difference in international performance between firms with and without sent-down experience CEO.

In year t, if a firm has a CEO with sent-down experience (*Sentdown* = 1), then the firm is in the treatment group, we have 396 firms in the treatment group. The potential control group is drawn from CEO without sent-down experience (*Sentdown* = 0) in year t. In year t-1, we use all the controlled variables in Model 2 of Table 3 to conduct a one-to-one PSM matching. We label the unmatched group as Group = U, while the matched group as Group = M.

Table 8A reports the differences in the means of the main control variables between the treated and control groups before and after matching. The results indicate that the control variables in the treated and control groups are significantly different before matching, while they are basically indistinguishable after matching, suggesting the efficiency of PSM approach.

**TABLE 8:** PSM regression: sent-down experience and enterprise internationalization.

(A) Covariate matching of variables					

Variables	Sign	Mean of treated	Mean of control	*t*-value	*p*-value
*Size*	U	22.9160	22.2000	10.55***	0.00
	M	22.9160	22.9630	–0.41	0.68
*Lev*	U	0.4837	0.4186	6.35***	0.00
	M	0.4837	0.4879	–0.31	0.76
*Top1*	U	0.4571	0.3009	6.50***	0.00
	M	0.4571	0.4470	0.29	0.78
*Board*	U	2.2710	2.2540	1.85*	0.06
	M	2.2710	2.2594	0.92	0.36
*Indep*	U	0.3756	0.3759	–0.13	0.90
	M	0.3756	0.3825	–1.73*	0.08
*Dual*	U	0.2677	0.3059	–1.6	0.11
	M	0.2677	0.2803	–0.4	0.69
*Tenure*	U	3.4760	3.4799	–0.07	0.94
	M	3.4760	3.4871	–0.16	0.88
*Edu*	U	2.4697	2.3589	1.2	0.23
	M	2.4697	2.5177	–0.38	0.70
*Gender*	U	0.8157	0.7576	2.62**	0.01
	M	0.8157	0.8182	–0.09	0.93
*Indusgrow*	U	0.0235	0.0391	–1.04	0.30
	M	0.0235	0.0382	–0.71	0.48
*IndusH*	U	0.8609	0.8718	–1.63	0.10
	M	0.8609	0.8510	1.03	0.30

(B) Regression of sent-down experience and enterprise internationalization using PSM matched samples				

	(1) Scope	(2) Scope	(3) Speed	(4) Speed

	Model 1	Model 2	Model 3	Model 4
*Sent-down*	3.411***	3.452***	0.148***	0.145***
	(4.94)	(5.07)	(6.23)	(6.19)
*Size*		–0.555		–0.004
		(–1.49)		(–0.39)
*Lev*		–0.610		–0.047
		(–0.33)		(–0.62)
*Top1*		0.570		0.023
		(0.64)		(0.83)
*Board*		–1.088		–0.001
		(–0.54)		(–0.01)
*Indep*		–0.353		–0.218
		(–0.08)		(–0.84)
*Dual*		0.875		0.049*
		(1.11)		(1.78)
*Tenure*		–0.472		–0.002
		(–1.39)		(–0.14)
*Edu*		0.182		–0.005
		(0.75)		(–0.67)
*Gender*		–0.950		–0.070
		(–0.53)		(–1.34)
*Indusgrow*		–0.280		–0.037
		(–0.32)		(–1.07)
*IndusH*		–4.958*		0.115
		(–1.68)		(1.01)
*_cons*	0.787	20.329***	0.791***	0.947***
	(0.39)	(2.80)	(5.13)	(3.04)
*N*	757	757	757	757
*adj. R^2^*	0.061	0.083	0.062	0.062

*t-Statistics in parentheses.*

*p < 0.1, **p < 0.05, ***p < 0.01.

We repeat the same regression as in Model 2 and 4 of Table 3 by using the matched sample in Table 8B. The results show that CEO sent-down experience remains significantly positive after the PSM treatment, denoting that the conclusions of our study are not affected by the CEO self-selecting problems. Thus, the result that CEO sent-down experience significantly positively affects the degree of internationalization of the focal firm is robust.

(4) Two-stage Heckman modeling. In the first stage of the analysis, we run a Probit analysis with *Sentdown* as dependent variable using the instrumental variable and the same control variables as in Column 2 of Table 3. We use the average Sentdown (*Average_Sentdown*) in the same year and the same industry as the instrumental variable. The logic is that if the specific industry tends to hire CEO with sent-down experience, the firm in the same industry has a higher probability to hire CEO with sent-down experience too. The results are presented in Column 1 of Table 9. The coefficients of *Average_Sentdown* are positive and significant at the 1% level, which is consistent with our conjecture that when an industry tends to hire a CEO with sent-down experience, the firm in the industry is more likely to do the same.

**TABLE 9:** Two-stage Heckman model.

	Step 1 Probit	Step 2 OLS	
	(1)	(2)	(3)

	Sentdown	Scope	Speed
*Average_Sentdown*	5.280***		
	(7.76)		
*Lambda*		2.659***	0.087***
		(3.50)	(3.16)
*Size*	0.218***	–1.210***	–0.027*
	(8.18)	(–2.99)	(–1.78)
*Lev*	0.087	–1.776	–0.100
	(0.49)	(–0.64)	(–0.99)
*Top1*	0.060	0.809	–0.002
	(0.89)	(0.86)	(–0.07)
*Board*	–0.472**	–3.853	–0.000
	(–2.51)	(–1.40)	(–0.00)
*Indep*	–1.273**	–1.643	0.383
	(–2.18)	(–0.17)	(1.10)
*Dual*	0.097	1.174	0.053
	(1.53)	(1.20)	(1.48)
*Tenure*	0.075**	–0.543	0.004
	(2.43)	(–1.33)	(0.28)
*Edu*	–0.020	0.170	–0.010
	(–1.03)	(0.63)	(–0.98)
*Gender*	0.139	–2.051	0.000
	(0.98)	(–1.62)	(0.00)
*ER*	–0.068	–0.213	–0.030
	(–0.72)	(–0.15)	(–0.59)
*EH*	–0.001	–5.973*	0.182
	(–0.01)	(–1.90)	(1.57)
*Constant*	–6.112***	50.338***	1.705***
	(–6.78)	(4.52)	(4.15)
*Year*	Yes	Yes	Yes
*Industry*	Yes	Yes	Yes
*N*	5862	5862	5862
*adj. R^2^/pseudo R^2^*	0.0976	0.112	0.109
*Likelihood ratio*	281.99***		

In the second stage, we calculate the predicted probability of CEO sent-down experience from the fitted values of the Probit model. Then we use this predicted probability to calculate an inverse Mill’s ratio to proxy for the likelihood of hiring a send-down experience CEO, which is indicated in the regression results as “Heckman’s lambda,” We then run the regression of *Scope and Speed* on *lambda* and all the control variables in Column 2 of Table 3. The results are presented in Column 2 and 3 of Table 9. The coefficients of *lambda* are positive as well as significant at the 1% level. The two-stage Heckman model yields qualitatively similar findings to those in Table 3.

## Conclusion and Discussion

### Main Findings

Drawing on the imprinting theory and upper echelons theory, we posit the positive effect of CEO early-life adversity experience on corporate internationalization. Using data on a sample of listed companies in China during the period 2007–2017, we obtained the following results. (1) CEO sent-down experience has a significantly positive effect on firm internationalization. (2) The impact of CEO sent-down experience on firm internationalization is stronger when the CEO has a higher level of organizational discretion (i.e., the firm has lower organizational inertia and more organization slack. (3) The positive effect of CEO sent-down experience on firm internationalization is stronger when the CEO has a higher level of institutional discretion (i.e., the firm is a non-SOE or operated in regions of a higher degree of marketization).

### Theoretical Contributions

Our study makes the following contribution. First, this study extends upper echelons research on the relationship between CEO characteristics and firm strategies and outcomes. While there is extensive literature examining the link between CEO characteristics and firm behaviors and a growing consensus that CEOs’ past experiences in life account for much of the variation in firm strategies and organization outcomes (Bernile et al., 2017; Sarfraz et al., 2020; Khan et al., 2021), largely overlooked is CEO early-life adversity experience. Studies in developmental psychology demonstrate that imprints from early-life experiences can be formative and enduring and manifest in various subsequent decisions (Caspi et al., 2005). Prior studies in management literature have investigated the role of CEO early-life poverty (Xu and Li, 2016), early-life disaster experience (O’Sullivan and Zolotoy, 2021) on firm strategies such as CSR and innovation, yet little is known about the impact of CEO early-life adversity instigated by social disorder. Using the send-down movement in china as the context of individual adversity, we analyze how CEO early-life adversity experience might affect their tolerance of uncertainty and change self-efficacy and then influence firm internationalization performance, enriching the stream of research on how senior executives’ early-life experiences influence firm strategies and outcomes. Although the sent-down experience has its specific historical context, and may not replicate elsewhere, this particular experience is possibly generalizable to any hardship experienced by adolescents that are out of their control. Thus, our study may help understand the role that such adversities, experienced by senior executives, play in firm internationalization, which adds to the research on CEO early-life in echoing the call from O’Sullivan and Zolotoy (2021).

Second, this study contributes to research on the antecedents of firm internalization from the perspective of higher echelons theory. The existing research mainly analyzes the effects of CEOs’ demographic characteristics (Camino Ramon-Llorens et al., 2017) or other explicit traits like CEO firm tenure (Jaw and Lin, 2009) or CEO educational attainment (Camino Ramon-Llorens et al., 2017; Chen Z. H. et al., 2020) on firm internationalization. Although these findings are insightful and help form the belief that CEOs’ experiences can play a significant role in the firm strategy of internationalization, the information revealed by the above-mentioned CEO characteristics can be mixed (for instance, CEO firm tenure can indicate both greater discretion and more inertia with regard to firm internationalization), which may lead to a contradictory effect on firm internationalization. Thus, research directly analyzing the impact of top managers’ early life experience or personalities can advance the research on how senior executives influence firm internationalization (Oesterle et al., 2016). By examining the link between CEO early-life adversity and firm internationalization, our study analyzes the impact of CEOs’ psychological traits imprinted by their early-life adversity and therefore provides a deeper understanding of the role of senior executives in firm internationalization.

Third, this study contributes to the existing studies on the historical event of the send-down movement. Our study examines internationally entrepreneurial exploration of the sent-down generation of entrepreneurs in China, which enriches research on send-down movement. Extant literature has studied the impact of the sent-down experience from historical and sociological perspectives on social trust (Liang and Li, 2014), political participation (Shi and Zhang, 2019), rural education (Chen Y. et al., 2020), etc., but there is little research on the impact of the sent-down experience on leaders at the economic level. This study supplements the stream of research by extending the impact of sent-down experience to firm internationalization performance.

### Managerial Implications

Firm internationalization strategy in emerging economies is important for its sustainable development. Despite many potential benefits of firm internationalization, there is a high level of uncertainty involved in the movement of goods and capital between different countries (Tihanyi et al., 2000). For example, changes in trade policy may pose significant risks to internationally oriented firms, such as the impact of former American President Trump’s protectionist policies and Brexit on the global trade structure, which is beyond managerial control. Therefore, it is critical to select CEOs appropriate to internationalization challenges.

In the executive selection candidates’ previous achievement and relevant experiences are normally highlighted. Yet we suggest that the board of firms intending to pursue international expansion also examine the candidates’ early-life experience when selecting and hiring CEOs as our findings indicate CEOs with early-life adversity experience could better promote firm internationalization. Executives who have overcome early-life adversity may have a higher level of self-efficacy in coping with change and uncertainty, which can be beneficial for leading internationalization explorations. Moreover, the study reveals that with a higher level of organizational and institutional discretion, the positive effect of CEO sent-down experience on firm internationalization performance is stronger. Therefore, enterprises aimed for internationalization may benefit more by conferring managerial discretion to CEOs with early-life adversity experiences to better play the positive effect of their experiences on firm internationalization.

### Limitations and Future Directions

With its theoretical and practical implications, the study has some limitations which should be considered in further research. First, due to data constraints, we did not test whether the mechanism underlying the positive relationship between CEO sent-down experience and firm internationalization performance is their higher level of tolerance of uncertainty and change self-efficacy imprinted by send-down campaign. Future studies may find appropriate methods to test the mechanism. Second, we did not explore the impact of the continuity of sent-down experience on firm internationalization. Although the send-down movements generally affected the urban youth born from 1946 to 1953, the extent varied a lot. Some of them endured the hardship for less than 2 years while others stayed much longer in the remote. It was not clear whether the duration of CEO early-life adversity experience influences the self-efficacy of the experiencers in coping with uncertainty and change, and thus firm internationalization performance. Future research may address these problems to provide a more comprehensive understanding of CEO adversity experience on firm internationalization.

## Data Availability Statement

Publicly available datasets were analyzed in this study. This data can be found here: www.gtarsc.com.

## Author Contributions

PZ: writing. YZ: processing data. KZ: providing revised advice. All authors contributed to the article and approved the submitted version.

## Conflict of Interest

The authors declare that the research was conducted in the absence of any commercial or financial relationships that could be construed as a potential conflict of interest.

## Publisher’s Note

All claims expressed in this article are solely those of the authors and do not necessarily represent those of their affiliated organizations, or those of the publisher, the editors and the reviewers. Any product that may be evaluated in this article, or claim that may be made by its manufacturer, is not guaranteed or endorsed by the publisher.

## Appendix

**Appendix A:** Industry-year distribution of the sample.

Industry	2007	2008	2009	2010	2011	2012	2013	2014	2015	2016	2017	Total
Agriculture, hunting, forestry, fishing	3	3	5	4	6	4	4	4	5	6	6	50
Mining	5	6	9	14	17	14	14	12	15	11	19	136
Manufacturing	111	134	162	198	322	419	483	529	596	728	895	4,577
Production and supply of electricity, heat, gas and water	0	2	2	3	6	5	4	3	1	2	2	30
Construction	5	8	12	8	13	15	13	16	16	21	31	158
Wholesale and retail	11	11	12	11	14	23	23	25	25	29	34	218
Transportation, warehousing and postal services	6	5	7	8	8	9	10	12	13	13	17	108
Hotel and catering	0	0	0	0	0	1	0	0	1	0	1	3
Information transmission, software and information technology services	6	5	13	19	23	20	28	30	36	61	69	310
Real estate	1	1	0	4	3	3	6	5	7	12	15	57
Leasing and commercial services	1	1	2	2	3	4	3	2	6	9	12	45
Scientific research and technical services	0	0	2	2	2	3	3	5	6	7	13	43
Water, environmental and public utility management	0	0	0	0	0	0	1	1	2	5	3	12
Residential services, repairs and other services	2	2	2	0	1	0	0	0	0	0	0	7
Education	0	0	0	0	0	0	0	1	1	2	4	8
Culture, sports and entertainment	0	0	1	1	1	3	3	4	10	11	15	49
Comprehensive	2	5	6	7	9	3	2	3	6	3	5	51
Total	153	183	235	281	428	526	597	652	746	920	1,141	5,862

*Industries are classified according to the code of China Securities Regulatory Commission (CSRC).*

**Appendix B:** Variable definitions.

Variable	Definitions	Data Source
**Dependent variables**		
Scope	The number of countries in which a firm established subsidiaries during its internationalization.	CSMAR
Speed	Five-year average growth ratio of the firm’s international revenue	CSMAR
**Independent variables**		
*Sentdown*	Dummy variable: it takes value of 1 if the CEO of a sample firm has Sent-down experience and 0 otherwise. The Sent-down experience. We collect the CEO names and birth year of all our sample firms, then we manually search for detailed resume information from Baidu Encyclopedia, Hexun News and the Encyclopedia of Financial Affairs and Encyclopedia. If CEO’s resume from 1968 to 1976 includes experiences such as “Zhiqing,” “farm,” “production and construction corps,” “commune,” We confirm his or her experience as possible Sent-down. We then match the resume information with the relevant information of China Youth Network to verify whether the ultimate controller has participated in the “going to the mountains and the countryside” movement. If the CEO has experienced the “going to the mountains and the countryside” movement, *Sent-down* dummy is defined as 1, and 0 otherwise.	Manually collected
*Sentdown2*	Dummy variable: it takes value of 1 if the chairman of the boarder in a sample firm has Sent-down experience and 0 otherwise.	Manually collected
**Moderating variables**		
Fage	Firm age was coded as the logarithmic number of years from the founding of a firm to the current year.	CSMAR
Slack	The ratio of equity/debt	CSMAR
SOE	A dummy variable which takes the value of one if the companies are state-owned, and zero otherwise.	CSMAR
Market	If a company is in a province/region with an index reading equal to or above the median value, the company falls into the high marketization group; it equals to one; otherwise, it belongs to the low marketization group, it equals to zero.	NERI Index of marketization of China’s provinces
**Control variables**		
Tenure	It is the natural log of the CEO tenure.	CSMAR
Edu	We use the education level to measure Edu variable. If the CEO with a Ph.D. degree, it equals to 5; If the CEO with a master degree, it equals to 4; If the CEO with a Bachelor degree, it equals to 3; If the CEO with a college degree, it equals to 2; otherwise equals to 1.	CSMAR
Gender	Dummy variable: 1 if CEO gender is male, and 0 otherwise	CSMAR
Size	Natural log of total assets	CSMAR
Lev	The ratio of total debt divided by total assets	CSMAR
Top1	The ratio of the number of shares held by the largest shareholder/total number of shares.	CSMAR
Dual	A dummy variable which takes the value of one if the chairperson of the board and CEO are the same person, and zero otherwise.	CSMAR
Board	The Logarithmic number of board members	CSMAR
indep	Proportion of independent directors	CSMAR
Indusgrow	The average growth in industry sales over the prior 5 years.	CSMAR
IndusH	It measures hypercompetitive environment, we use the concentration of sales and is computed as EH = 1-$\sum_{i=1}^{n}h_{i}^{2}$; where hi is the percentage sales of industry i.	CSMAR
